# A Mechanism for Apoptotic Effects of a Planar Catechin Analog on Cancer Cells

**DOI:** 10.3390/molecules29184467

**Published:** 2024-09-20

**Authors:** Hiromu Ito, Yoshimi Shoji, Ken-ichiro Matsumoto, Kiyoshi Fukuhara, Ikuo Nakanishi

**Affiliations:** 1 Quantum RedOx Chemistry Team, Quantum Life Spin Group, Institute for Quantum Life Science (iQLS), National Institutes for Quantum Science and Technology (QST), Inage-ku, Chiba 263-8555, Japan; shoji.yoshimi@qst.go.jp (Y.S.); fukuhara@pharm.showa-u.ac.jp (K.F.); 2 Quantitative RedOx Sensing Group, Department of Radiation Regulatory Science Research, Institute for Radiological Science (NIRS), National Institutes for Quantum Science and Technology (QST), Inage-ku, Chiba 263-8555, Japan; matsumoto.kenichiro@qst.go.jp; 3 Division of Organic and Medicinal Chemistry, Showa University School of Pharmacy, Shinagawa-ku, Tokyo 142-8555, Japan

**Keywords:** planar catechin, gastric cancer cells, reactive oxygen species, NF-κB, apoptosis

## Abstract

Catechin is one of the representative antioxidants that shows physiological activities such as an anti-cancer effect. We have developed a chemically modified catechin analog possessing a planar structure, which shows an enhanced radical-scavenging activity as well as inhibitory effects on the proliferation and migration of cancer cells, compared to the parent (+)-catechin. In this study, the mechanism for cancer cell inhibition by the planar catechin was partly elucidated using a gastric cancer cell line. The planar catechin treatment induced an enhanced expression of an apoptotic marker, cleaved caspase-3, in addition to the mitigation of the intracellular accumulation of reactive oxygen species (ROS) and NF-κB expression. Furthermore, γH2AX, a marker of double-strand breaks in DNA, was also induced by the planar catechin treatment in a dose-dependent manner. These findings suggest that the removal of ROS by the planar catechin with a higher antioxidant ability executed NF-κB suppression and/or the planar catechin-injured DNA, leading to the induction of apoptosis in cancer cells.

## 1. Introduction

Cancer is one of the major causes of death worldwide today, and many kinds of medical technics have been explored to find an effective therapy. Surgery, anti-cancer drugs, and radiotherapy have been utilized to achieve the complete remission of cancer. However, these treatments often cause side effects and reduce quality of life for patients. Therefore, an effective alternative method with less side effects is required.

Cancer cells produce elevated levels of reactive oxygen species (ROS) as a by-product of increased metabolism, leading to cancer development and maintenance [1]. ROS are also related to the malignant progression of cancer, such as metastasis, migration, and invasion [1,2,3]. We previously reported that ROS derived from mitochondria were overgenerated in cancer cells and that the overexpression of mitochondrial ROS-scavenging enzymes suppressed cancer cellular migration and invasion [4]. Since the alleviation of ROS levels in cancer cells inhibits cancer cellular activity, the administration of antioxidants could be an effective way to treat cancer cells.

Catechins in green tea are known to be an antioxidant, and they also show an anti-cancer effect [5]. However, the bioavailability of catechin in the human body is low, and improvements in bioavailability, along with another method, are needed to achieve an effective treatment [6]. We have previously developed a catechin analog with a planar structure (Figure S1), and the planar catechin showed a 10-fold larger second-order rate constant than (+)-catechin for the reaction with 2,2-diphenyl-1-picrylhydrazyl radical (DPPH^•^) [7,8]. In addition, the planar catechin showed enhanced protective effects against apoptosis induced by X-ray irradiation compared to (+)-catechin because of the higher radical-scavenging activity and lipophilicity [8]. We also reported that the planar catechin showed higher cancer cell-dominant cytotoxicity and had a decreasing effect on the mitochondrial membrane potential compared to normal cells [9]. Furthermore, the cancer cell-dominant inhibition of cell migration by the planar catechin has also been reported [10]. However, a detailed mechanism for cancer cellular inhibition by the planar catechin is still unclear. In this study, the elucidation of a part of the mechanism for cancer cellular inhibition by the planar catechin was examined.

## 2. Results

### 2.1. Enhancement of Cleaved Caspase-3 Expression

**Figure 1:**
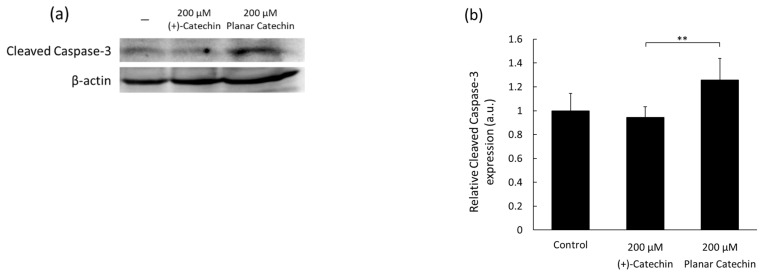

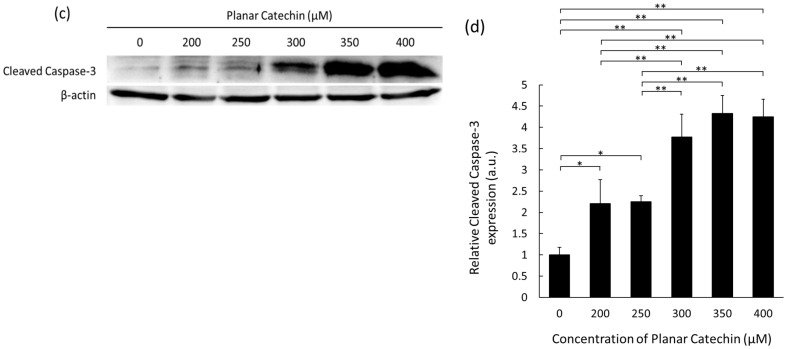
The expression levels of cleaved caspase-3 after treatment of (+)-catechin or the planar catechin were analyzed by Western blotting. (**a**) Representative blotting images of cleaved caspase-3 and β-actin after treatment of 200 μM (+)-catechin or the planar catechin. (**b**) Relative cleaved caspase-3 expression after treatment of 200 μM (+)-catechin or the planar catechin. *n* = 5, mean ± S.D., ** *p* < 0.01, Dunn-Bonferroni. (**c**) Representative blotting images of cleaved caspase-3 and β-actin with changing concentrations of the planar catechin. (**d**) Relative cleaved caspase-3 expression with changing concentrations of the planar catechin. *n* = 3, mean ± S.D., * *p* < 0.05, ** *p* < 0.01, Tukey’s HSD.

Caspase-3 is known as a major executioner of apoptosis and is cleaved and activated by initiator caspases [11,12]. The representative blotting band images of cleaved caspase-3 and β-actin were shown, and the expression level of cleaved caspase-3 was significantly elevated by the planar catechin treatment compared to the control and (+)-catechin treatment (Figure 1a,b). We also examined the dose-dependent effects of the planar catechin in terms of the expression of cleaved caspase-3 and demonstrated that the dose-dependent increase in cleaved caspase-3 expression was caused in RGK1 cells by the treatment of the planar catechin (Figure 1c,d), but there was no expression with the (+)-catechin treatment (Figure S2). These results indicate that the planar catechin is a more effective apoptotic inducer compared to (+)-catechin and that the effect is enhanced dose-dependently.

### 2.2. Dose-Dependent Elevation in γH2AX Expression

**Figure 2:**
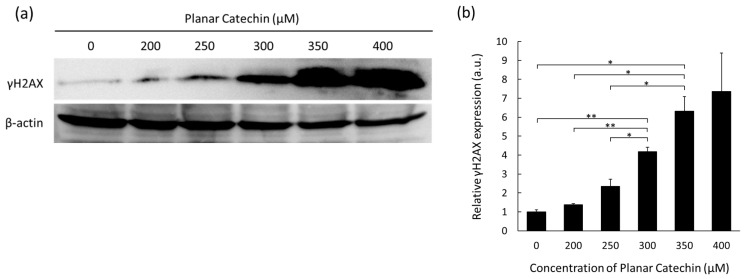
The expression levels of γH2AX after treatment of the planar catechin were analyzed by Western blotting. (**a**) Representative blotting images of γH2AX and β-actin with changing concentrations of the planar catechin. (**b**) Relative γH2AX expression with changing concentrations of the planar catechin. *n* = 3, mean ± S.D., * *p* < 0.05, ** *p* < 0.01, Games-Howell.

When double-strand breaks are introduced into the DNA, phosphorylated histone H2AX on serine 139 (γH2AX) is expressed [13]. Thus, the effects of the planar catechin on DNA damage were examined. Figure 2a shows representative images of Western blotting bands of γH2AX and β-actin. The expression levels of γH2AX were enhanced in a dose-dependent manner of the planar catechin. The results of the band intensity measurements also show a dose-dependent increase in γH2AX (Figure 2b). These results indicate that the planar catechin induced DNA injury in cancer cells in a dose-dependent manner. 

### 2.3. Decrease in Intracellular ROS by the Planar Catechin Treatment

**Figure 3:**
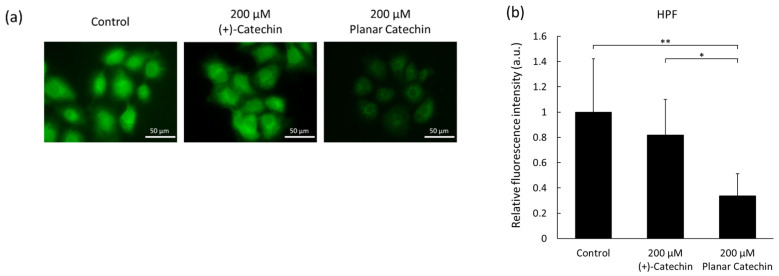
Intracellular ROS were detected with HPF. (**a**) Representative microscopic images of HPF in cells. Scale bar: 50 μm. (**b**) Relative fluorescence intensities of HPF in microscopic analysis. *n* = 6, mean ± S.D., * *p* < 0.05, ** *p* < 0.01, Dunn-Bonferroni.

Intracellular ROS levels in cancer cells were estimated by microscopy, and the effects of the planar catechin treatment were examined. The fluorescence images of HPF in cells were obtained, and relatively strong fluorescence was detected in the control and (+)-catechin-treated cells. On the contrary, the planar catechin treatment decreased the intracellular fluorescence (Figure 3a,b). As another method, flowcytometric analysis was performed, and the fluorescence intensity of HPF in cells treated with the planar catechin decreased compared to the control and (+)-catechin-treated cells (Figure S3). Similar results were observed in both microscopic and flowcytometric analyses in the detection of intracellular HPF fluorescence. These results indicate that cancer cells produced high amounts of ROS and that the planar catechin treatment effectively decreased ROS generation in cancer cells.

### 2.4. Decrease in NF-κB and IκBα by the Planar Catechin Treatment

**Figure 4:**
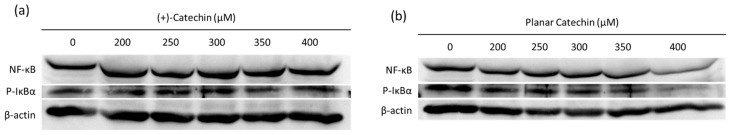

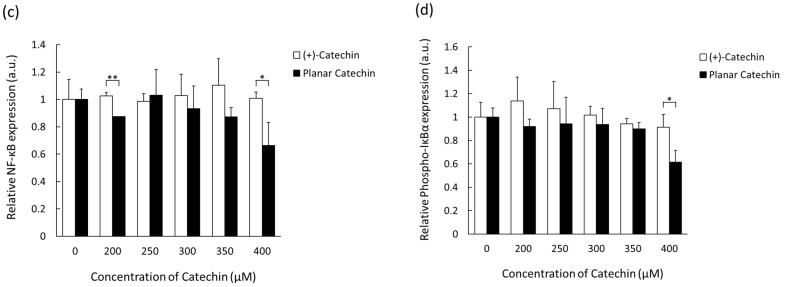
The expression levels of NF-κB and P-IκBα after treatment of (+)-catechin or the planar catechin were analyzed by Western blotting. (**a**) Representative blotting images of NF-κB, P-IκBα, and β-actin with changing concentrations of (+)-catechin. (**b**) Representative blotting images of NF-κB, P-IκBα, and β-actin with changing concentrations of the planar catechin. (**c**) Relative NF-κB expression with changing concentrations of (+)-catechin or the planar catechin. (**d**) Relative phosphorylated IκBα expression with changing concentrations of (+)-catechin or the planar catechin. *n* = 3, mean ± S.D., * *p* < 0.05, ** *p* < 0.01, Student’s *t*-test or Mann-Whitney *U* test.

NF-κB is a transcription factor activated through the phosphorylation and degradation of IκBα [14]. ROS such as hydrogen peroxide, superoxide, and singlet oxygen are a mediator of NF-κB activation, and the activation of the NF-κB-signaling pathway is related to the malignancy of cancer cells [15,16,17,18]. The effects of the planar catechin with strong antioxidant capacity on the expression of NF-κB and IκBα were studied. Figure 4a,b show representative blotting images of NF-κB and phosphorylated IκBα (P-IκBα) after the treatment of (+)-catechin or the planar catechin. Figure 4c,d show the relative expressions of NF-κB and P-IκBα after the treatment of (+)-catechin or the planar catechin. The planar catechin treatment induced a dose-dependent reduction in both NF-κB and P-IκBα expressions, particularly significant at 400 μM, while no change was observed in the case of the treatment with (+)-catechin. These findings suggest that the planar catechin inhibits the expression and activation of NF-κB by the suppression of the phosphorylation of IκBα, leading to the repression of cancer cellular activities.

## 3. Discussion

The planar catechin analog possessing a strong antioxidant capacity showed a remarkable decrease in cell viability in cancer cells, as we previously reported [9]. In this study, the expression of caspase-3 was enhanced by the planar catechin treatment (Figure 1), and the type of cancer cell death would be dictated as apoptosis since caspase-3 is an apoptosis marker. Another type of catechin was also reported to induce cancer cell death through apoptosis [19]. The planar catechin was reported to have a higher scavenging activity against DPPH^•^ than (+)-catechin and was demonstrated to remove intracellular ROS (Figure 3a,b) [8]. We also reported that the planar catechin reduced mitochondrial membrane potential, and the induction of apoptosis by the planar catechin would be associated with the affection for mitochondria [9]. Mitochondria are the major producer of ROS, and mitochondrial ROS may be related to oncogenesis [20,21]. In cancer cells, ROS are generated at higher levels than in normal cells, and increased ROS are involved in cancer development and progression [22,23]. NF-κB plays a pivotal role in cancer cellular activities and is involved in cell invasion and metastasis [24]. The planar catechin would contribute to inhibiting cancer cellular activities through the suppression of NF-κB expression and activation since the expression of NF-κB actually decreased (Figure 4). In fact, the migration activity of cancer cells was clearly suppressed by the planar catechin treatment, as we reported previously [10].

Although the removal of ROS could inhibit cancer cellular activities, excess amounts of ROS also damage proteins, nucleic acids, and organelles, which can activate the processes of cell death such as apoptosis [25]. Cisplatin, one of the major chemotherapeutic drugs, is reported to promote mitochondrial ROS generation and induce cellular apoptosis [26]. On the other hand, high-performance antioxidants can be easily oxidized, and the antioxidants themselves have a potential to be new reactive pro-oxidants. Nakagawa et al. reported that catechins are converted from antioxidants to pro-oxidants in the presence of Cu (II) [27]. These pro-oxidants derived from antioxidants may also be related to cell injury.

Cell apoptosis can be induced by not only ROS but also DNA injury. As shown in Figure 2, the planar catechin indeed showed the effects of DNA injury. Tea catechins including (+)-catechin are also reported to interact with DNA [28]; however, no acute DNA injury in RGK1 cells was observed with (+)-catechin treatment for 24 h under the same treatment condition as the case of the planar catechin treatment (Figure S4). Meanwhile, quercetin is one of the flavonoids with a similar structure to catechin. Quercetin has a higher antioxidant capacity comparable to catechins and directly interacts with DNA, leading to apoptosis in cancer cells via mitochondria [29]. Since quercetin also has a planar structure, which makes it possible to intercalate to DNA, the planar structure may be an important factor for DNA injury in cancer cells [30]. The properties and results reported in this study suggest that the planar catechin may be a promising agent for cancer therapy. We are now further investigating the effects of the planar catechin using an in vivo model.

In conclusion, the catechin analog possessing the planar structure with a higher antioxidant capacity than (+)-catechin showed intracellular ROS scavenging ability, leading to the suppression of NF-κB and inhibition of cell activities in cancer cells. In addition to ROS removal, we suggest that the induction of DNA injury by the planar catechin caused cancer cellular apoptosis.

## 4. Materials and Methods

### 4.1. Cell Culture

A rat gastric cancer cell line, RGK1, was cultured in DMEM supplemented with 10% fetal bovine serum (Thermo Fisher Scientific, Inc., Waltham, MA, USA) and 1% penicillin/streptomycin (Thermo Fisher Scientific, Inc.). Cells were cultured in a humidified condition under 5% CO_2_ at 37 °C.

### 4.2. Western Blotting Analysis

The expression levels of cleaved caspase-3, γH2AX, a transcription factor NF-κB, and phosphorylated IκBα after the treatment of (+)-catechin or the planar catechin were examined. Cells were treated with 0, 200, 250, 300, 350, and 400 μM (+)-catechin or the planar catechin, referring to the previous report, and incubated for 24 h [9]. The proteins were collected and mixed with NuPAGE™ LDS Sample Buffer (Thermo Fisher Scientific, Inc.) containing 5% (*v*/*v*) 2-mercaptoethanol (FUJIFILM Wako Pure Chemical Corporation, Osaka, Japan) and heated at 95 °C for 5 min. Samples of 20 μg of protein were applied to 10% (*w*/*v*) or 18% (*w*/*v*) polyacrylamide gels and electrophoresed with 200 V setting voltage in flesh running buffer containing 25 mM Tris, 192 mM glycine, and 0.1% (*w*/*v*) sodium dodecyl sulfate. The proteins in the gels were electrically transferred onto polyvinylidene difluoride (PVDF) membranes (Merck Millipore, Burlington, MA, USA) with AquaBlot™ Transfer Buffer (FUJIFILM Wako Pure Chemical Corporation). Membranes were incubated with PVDF Blocking Reagent for Can Get Signal^®^ (TOYOBO Co., Ltd., Osaka, Japan) for 1 h and treated with cleaved caspase-3 antibody, γH2AX antibody, and NF-κB antibody (Cell Signaling Technology, Inc., Danvers, MA, USA) diluted with Can Get Signal^®^ Immunoreaction Enhancer Solution 1 (TOYOBO Co., Ltd.) at 1:500 and incubated at 4 °C overnight. The membranes were washed with PBS-T and incubated with horseradish peroxidase (HRP)-linked secondary antibodies at 25 °C. The membranes were washed with PBS-T and treated with Immobilon Forte Western HRP substrate (Merck Millipore, Burlington, MA, USA), and the chemical luminescence was detected with the Lumino Image Analyzer Las 4000 mini (FUJIFILM Corporation, Tokyo, Japan). β-actin was also detected as a sample loading control. In the case of phosphorylated IκBα detection, β-actin antibodies were removed with Western BLoT Stripping Buffer (Takara Bio Inc., Shiga, Japan) for 30 min and phospho-IκBα was detected with the antibody (Cell Signaling Technology, Inc.).

### 4.3. Microscopic Analysis of Intracellular ROS

Intracellular ROS levels in cells were examined by microscopic analysis after the treatment of (+)-catechin or the planar catechin. Cells were seeded on a glass bottom dish at a density of 5 × 10^4^ cells/dish and incubated overnight. The medium was replaced with flesh one containing 200 μM (+)-catechin or the planar catechin and incubated for 24 h. The medium was discarded, and cells were incubated with 10 μM hydroxyphenyl fluorescein (HPF) (Goryo Chemical, Inc., Hokkaido, Japan), which reacts with highly reactive oxygen species in cells and emits green fluorescence for 30 min at 37 °C. HPF fluorescence was visualized by a BZ-8000 fluorescence microscope (KEYENCE CORPORATION, Osaka, Japan) with a Plan Fluor 20× objective lens and a GFP filter.

### 4.4. Statistical Analysis

Statistical analysis was performed with SPSS statistics 28 software (International Business Machines Corporation, Armonk, NY). Shapiro-Wilk test was used for the assessment of data normality. Tukey’s HSD, Games-Howell or Dunn-Bonferroni test was used to examine the significant differences for more than three data sets and Student’s *t*-test or Mann-Whitney *U* test was used for the comparison of two data sets. All data are presented as mean ± standard deviation.

## Data Availability

Data are contained within the article.

## Supplementary Materials

The following supporting information can be downloaded at: https://www.mdpi.com/article/10.3390/molecules29184467/s1, Figure S1: Chemical structures of (+)-catechin and the planar catechin; Figure S2: The expression levels of cleaved caspase-3 after treatment of (+)-catechin; Figure S3: Flowcytometric analysis of HPF fluorescence in cancer cells; Figure S4: The expression levels of γH2AX after treatment of (+)-catechin.
