# Supplementary material for: A Mechanism for Apoptotic Effects of a Planar Catechin Analog on Cancer Cells
Source: Molecules. 2024 Sep 20;29(18):4467. doi: 10.3390/molecules29184467 (PMC11433776; doi:10.3390/molecules29184467)
Supplement: Supplementary file 1 [file molecules-29-04467-s001.zip › molecules-3162394-supplementary.pdf]

Supporting Information

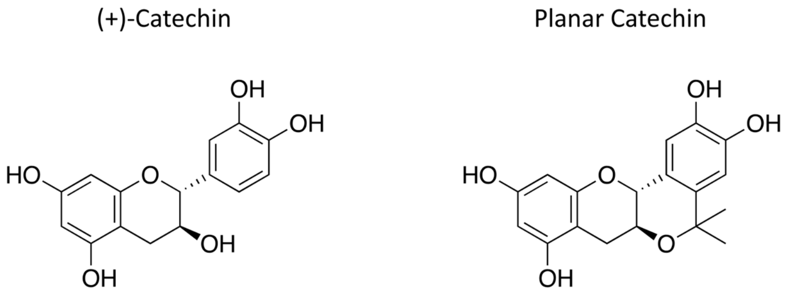

Figure S1. Chemical structures of (+)-catechin and the planar catechin.

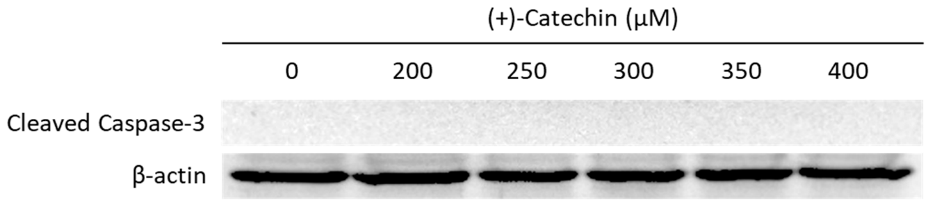

Figure S2. The expression analysis of cleaved caspase-3 and β-actin with various concentrations of (+)-catechin by Western blotting.

Flowcytometric Analysis of Intracellular ROS

Flowcytometric measurement was also carried out to examine the intracellular ROS levels in RGK1 cells after treatment of (+)-catechin or the planar catechin. Cells were seeded on a 100 mm dish at a density of 5×10^5 cells/dish and incubated overnight. The medium was replaced with flesh one containing 200 μM (+)-catechin or the planar catechin and incubated for 24 h. The medium was discarded, and cells were washed with PBS and detached by trypsinization. The cells were centrifuged at $200 \times g$ for 3 min and the supernatant was removed. The cells were treated with 10 μM HPF for 30 min at 37 °C, and after incubation, centrifuged at $200 \times g$ for 3 min and the supernatant was removed. The cells were suspended with PBS and centrifuged again. After the supernatant was discarded, the cells were resuspended with PBS and the HPF fluorescence was analyzed by FACSVerse™ (Becton, Dickinson and Company, Franklin Lakes, NJ) with FITC filter.

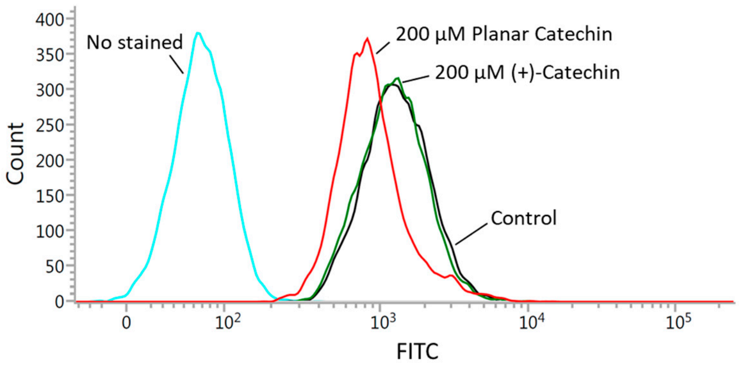

Figure S3. Flowcytometric analysis of HPF fluorescence in cancer cells.

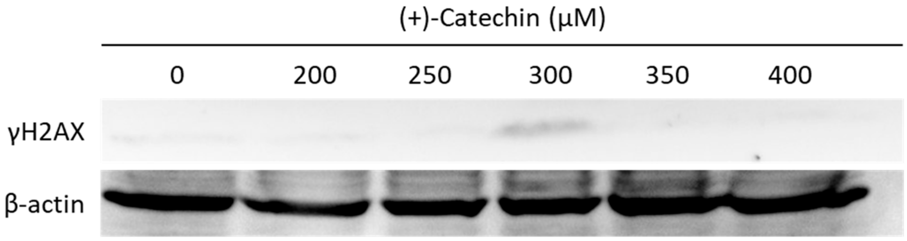

Figure S4. The expression analysis of γH2AX and β-actin with various concentrations of (+)-catechin by Western blotting.
